# Case Report: Pigmented Villonodular Synovitis in a Patient With Sjögren's Syndrome

**DOI:** 10.1111/1756-185x.70392

**Published:** 2025-08-13

**Authors:** Ting‐Yu Chang, Chun‐Cheng Liao, Tao‐An Chen, Deng‐Ho Yang, Chia‐Wen Kuo

**Affiliations:** ^1^ Department of Family Medicine Taichung Armed Forces General Hospital Taichung Taiwan; ^2^ School of Medicine National Defense Medical University Taipei Taiwan; ^3^ Institute of Medicine Chung Shan Medical University Taichung Taiwan; ^4^ Department of Medical Education and Research Taichung Armed Forces General Hospital Taichung Taiwan; ^5^ Department of Pathology Taichung Armed Forces General Hospital Taichung Taiwan; ^6^ Division of Rheumatology/Immunology/Allergy, Department of Internal Medicine Taichung Armed Forces General Hospital Taichung Taiwan; ^7^ Division of Rheumatology/Immunology/Allergy, Department of Internal Medicine Tri‐Service General Hospital, National Defense Medical University Taipei Taiwan; ^8^ Department of Medical Laboratory Science and Biotechnology Central Taiwan University of Science and Technology Taichung Taiwan; ^9^ Institute of Biomedical Science National Chung‐Hsing University Taichung Taiwan; ^10^ College of Life Sciences National Chung Hsing University Taichung Taiwan; ^11^ Division of Nephrology, Department of Internal Medicine Taichung Armed Forces General Hospital Taichung Taiwan

Dear Editor,

1

Pigmented villonodular synovitis (PVNS) is a rare but locally aggressive proliferative lesion that arises in the synovium, tendon sheaths, and bursae. According to the 2020 WHO Classification of Tumours of Soft Tissue and Bone, PVNS is now classified as diffuse‐type tenosynovial giant cell tumor (diffuse‐TGCT) [1].

PVNS primarily affects large joints, most commonly the knee, followed by the hip, ankle, and shoulder. It is characterized by nonspecific symptoms, such as joint pain, swelling, stiffness, and limited range of motion, which often leads to misdiagnosis as other inflammatory or degenerative joint disorders [1].

While the exact etiology of PVNS remains unclear, it is believed to involve neoplastic and inflammatory components. Recent studies suggest that a neoplastic process driven by a translocation involving colony‐stimulating factor 1 (CSF1) and its receptor (CSF1R) leads to abnormal macrophage recruitment and infiltration of other inflammatory cells, thereby contributing to disease progression. This process leads to chronic inflammation, synovial proliferation, and cartilage destruction. The deposition of hemosiderin resulting from recurrent intra‐articular bleeding contributes to increased tissue damage and fibrosis. Risk factors for progression include delayed diagnosis, incomplete surgical resection, recurrent synovial inflammation, and disease recurrence [2, 3].

Hemarthrosis detected through joint aspiration can be a crucial diagnostic clue for PVNS. Magnetic resonance imaging (MRI) is essential, typically revealing a well‐defined lesion with hemosiderin deposition, characterized by low signal intensity on T2‐weighted sequences. Histopathologically, PVNS is characterized by mononuclear cells, multinucleated giant cells, and hemosiderin‐laden macrophages [4, 5].

The treatment primarily involves surgical resection, often through arthroscopic synovectomy; however, the recurrence rate remains high. In some cases, adjuvant therapies—such as radiotherapy or targeted molecular treatments such as tyrosine kinase inhibitors (e.g., pexidartinib)—may be considered to reduce the risk of recurrence [2, 6].

This case report describes a patient with an underlying diagnosis of Sjögren's syndrome who was diagnosed with PVNS based on pathological findings from a total knee replacement (TKR). We aim to highlight the key considerations in its diagnosis and management.

A 62‐year‐old Taiwanese woman presented with a 5‐year history of persistent right knee pain and limited range of motion. She is a chronic hepatitis B carrier under regular monitoring. She does not smoke or drink. She has no significant family history. Initially, she was diagnosed with a knee effusion in her right knee and underwent arthroscopy in 2017. However, her symptoms gradually worsened, significantly impairing daily life. In 2022, she sought medical care at our orthopedic outpatient department, where a varus deformity of the right knee was observed. The physical examination revealed a positive patellar grind test, limited range of motion, and flexion contractures in both knees. The radiograph revealed joint space narrowing and obliteration in the right knee, with marginal spur formation. Joint aspiration yielded bloody effusion (Red blood cells: 591 700/μL, reference range: < 100/μL). The initial diagnosis was osteoarthritis of the right knee.

Following discussion, a TKR was performed, during which PVNS was suspected. The histopathological examination revealed hyperplastic synovium with papillary projections, composed of mononuclear cells, hemosiderin‐laden macrophages, and occasional multinucleated giant cells, consistent with PVNS, with no morphological evidence of malignancy (Figure 1).

**FIGURE 1:**
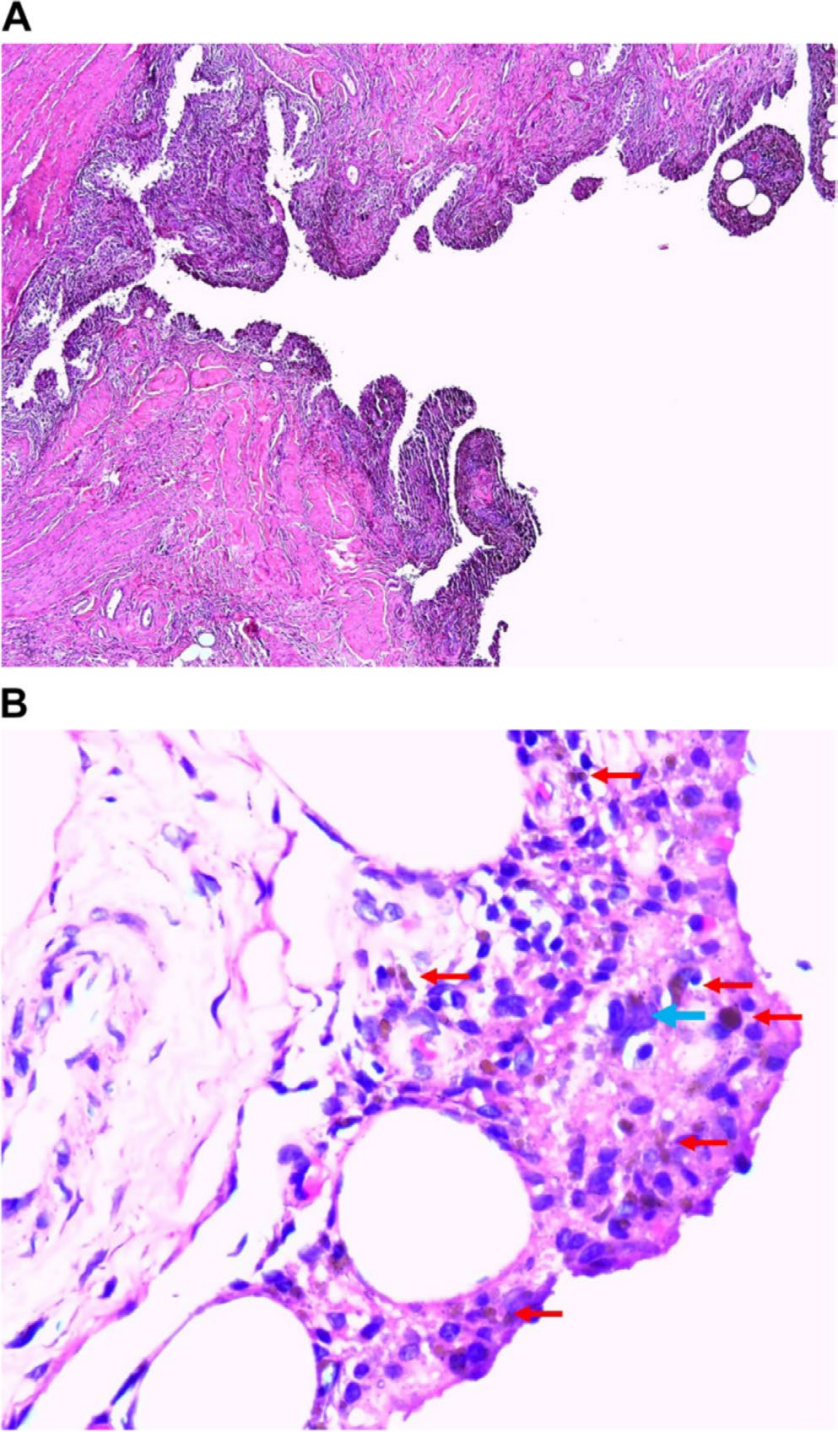
Synovial biopsy microscopy image. (A) H&E stain, 40×: hyperplastic synovium with villous papillary projections. (B) H&E stain, 400×: admixture of mononuclear tumor cells, hemosiderin‐laden macrophages (red arrows), and multinucleated giant cells (blue arrow) infiltrating the synovium.

2

Despite the surgery, her symptoms persisted. As a result, she underwent two revision surgeries in July and September 2023. Dry eyes and mouth were also observed during follow‐up. Owing to postoperative fatigue, a preference for conservative treatment, and new symptoms, she was referred to the rheumatology clinic in January 2024. Laboratory results showed normal erythrocyte sedimentation rate, C‐reactive protein, and white blood cell (WBC) count. The autoantibody panel was performed and showed positive anti‐double‐stranded deoxyribonucleic acid antibodies (40 IU/mL/; positive: > 35 IU/mL), positive anti‐Sjögren's‐syndrome‐related antigen A autoantibodies (11 IU/mL; positive: > 10 IU/mL), and negative results for human leukocyte antigen B27, anti‐nuclear antibodies, and anti‐Sjögren's‐syndrome‐related antigen B autoantibodies. The clinical impression was Sjögren's syndrome. Subsequently, prednisone (5 mg twice daily), hydroxychloroquine (200 mg twice daily), and leflunomide (10 mg once daily) were prescribed for immunotherapy.

Although she underwent additional surgeries because of persistent symptoms, the interval between procedures increased significantly after initiating medical treatment (Figure 2). The primary method for monitoring the stability of PVNS is imaging, particularly MRI. However, in this case, MRI could not be performed due to the patient's history of total knee replacement (TKR) surgery, and conventional X‐rays have limited sensitivity in detecting soft tissue lesions. Regarding blood markers, the literature suggests that the neutrophil‐lymphocyte ratio (NLR) and CRP can be used for prognostic assessment [7]. In this case, the patient received immunotherapy to control systemic inflammation with normal levels of ESR and CRP, and active recurrent PVNS may be suppressed. Additionally, the patient's range of motion was measured and remained between grade 0 and 1, and there was also improvement in the Visual Analog Scale (VAS) pain score, which stabilized at 2–3 points. She remains under continuous monitoring and follow‐up in the orthopedics and rheumatology clinics.

**FIGURE 2:**
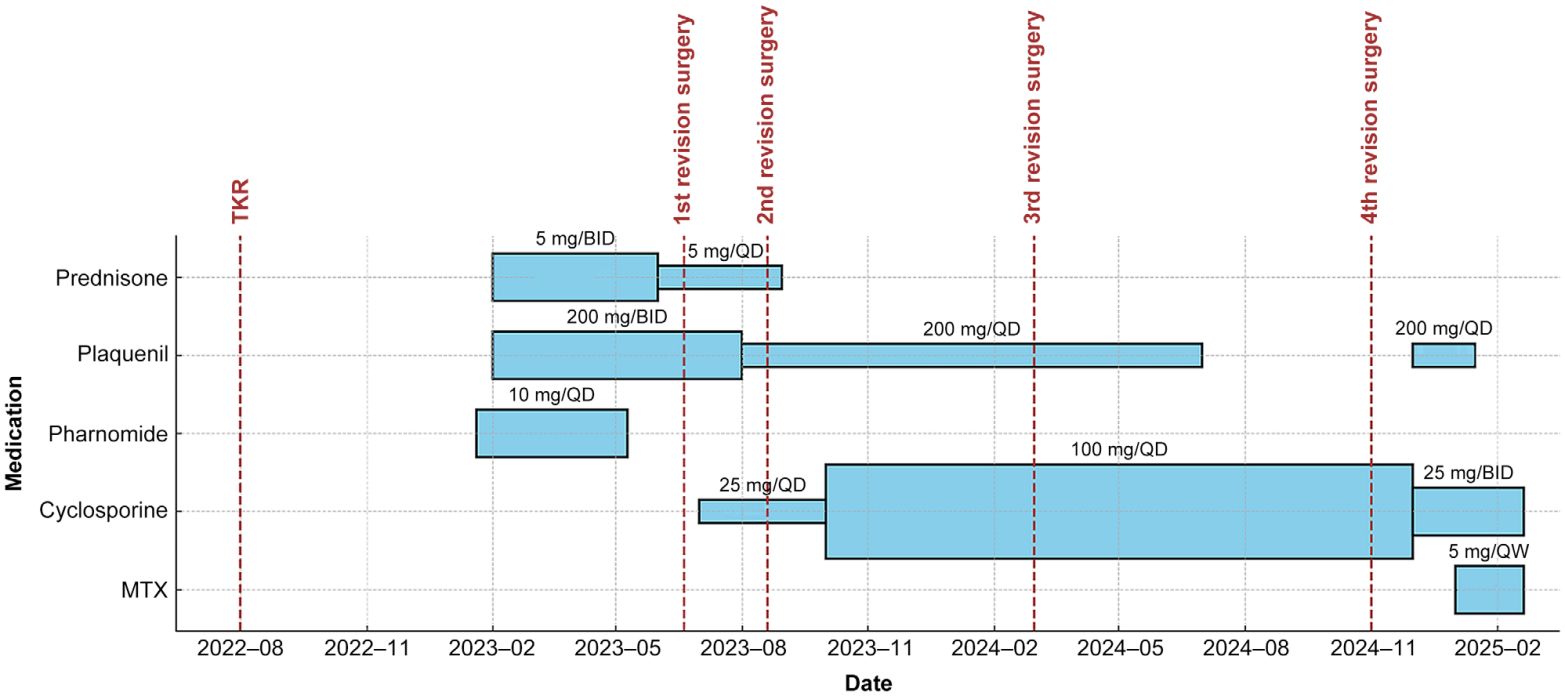
Timeline of medication and surgery for this case.

3

Initially, the patient was diagnosed with osteoarthritis based on chronic knee pain, joint stiffness, and limited range of motion—symptoms commonly seen in degenerative joint disease. The differential diagnosis for monoarthritis is broad, including septic arthritis, crystal‐induced arthritis, hemarthrosis (from trauma or pigmented villonodular synovitis, PVNS), tumors, seronegative spondyloarthropathy, and osteoarthritis. Arthrocentesis is a key diagnostic step, distinguishing infection (WBC > 50 000/mm^3^ and positive culture), crystal‐induced inflammation, hemorrhagic conditions like PVNS, and degenerative causes (low WBC count) [8]. Although joint aspiration in this case revealed a bloody effusion (RBC: 591 700/μL), which should have raised suspicion for PVNS, the rarity of PVNS (about 1.8 per million people per year) meant it was not initially prioritized in the diagnostic workup [9]. Imaging studies are equally important. While plain radiographs showed joint space narrowing—findings that are often ascribed to osteoarthritis—closer examination later revealed more severe lateral compartment involvement, an atypical feature for degenerative disease. The lack of advanced imaging, particularly MRI, further limited early recognition of PVNS. Also, immunohistochemistry and molecular markers related to PVNS, such as CSF1 and CD68, are not routinely performed at our institution. Cost considerations and the perceived unlikelihood of rare conditions like PVNS meant MRI was not performed, and the diagnosis was made intraoperatively during TKR when synovial pathology was established.

This case emphasizes several important points for clinical practice: persistent monoarthritis with hemorrhagic effusion, rapid joint degeneration, or atypical radiographic features should raise suspicion for PVNS. In these situations, MRI—despite its cost—can significantly improve diagnostic accuracy. Enhanced awareness of PVNS and multidisciplinary discussions, along with careful review of imaging and aspiration findings, are essential to avoid missed or delayed diagnoses. Following these principles may help clinicians reduce missed PVNS cases and improve both perioperative planning and long‐term patient outcomes.

The patient had a history of Sjögren's syndrome, a systemic autoimmune disease. The relationship between Sjögren's syndrome (SS) and pigmented villonodular synovitis (PVNS) remains speculative, but several immunopathological overlaps have been observed. Both diseases are characterized by marked immune cell infiltration—CD4+ and CD8+ T lymphocytes in SS and predominantly CD68+ macrophages (with some T cells) in PVNS. Chronic inflammatory environments in each condition include elevated cytokines such as IL‐6, TNF‐α, and IL‐1β, suggesting that persistent immune activation in SS may create a milieu conducive to synovial proliferation or may influence the recurrence of PVNS [1, 10]. Therefore, active inflammation can be observed during the disease of SS, and this inflammation may be associated with recurrent PVNS.

The pathogenesis of PVNS is primarily driven by the overexpression of colony‐stimulating factor 1 (CSF1) and activation of its receptor (CSF1R), resulting in abnormal recruitment and proliferation of monocyte/macrophage lineage cells. Recent research has identified IL‐34 as an alternative ligand for CSF1R, independent of CSF1 [11, 12]. Notably, IL‐34 has been found to be overexpressed in the inflamed salivary glands of patients with primary Sjögren's syndrome [13]. This overexpression is associated with the expansion of pro‐inflammatory monocyte populations in affected tissue. These findings indicate that the CSF1/CSF1R/IL‐34 axis may represent a shared pathological pathway between SS and PVNS.

Although immunohistochemistry for CSF1R was not performed in this case, current evidence underscores its central role not only in PVNS but also in various autoimmune diseases, such as rheumatoid arthritis, SLE, and psoriatic arthritis [14]. There are isolated reports of PVNS occurring in patients with systemic autoimmune diseases, including SS, SLE, RA, and psoriatic arthritis, which supports the possibility of a link; though causality has not been established [15, 16, 17, 18].

Whether PVNS can result directly from SS‐related chronic inflammation remains unproven. To establish a causal association, future studies are needed—incorporating epidemiological analysis, molecular profiling (including assessment of CSF1R and its ligands), and comparative studies of patients with overlapping syndromes. Ultimately, recognition of this potential overlap is important for timely diagnosis and may have implications for the use of systemic immunosuppressive therapies, as our case demonstrated a prolonged interval between surgical interventions after immunotherapy.

## Author Contributions

Conceptualization, T.‐Y.C., D.‐H.Y., and C.‐W.K.; methodology, D.‐H.Y., C.‐W.K., and C.‐C.L.; investigation, T.‐Y.C. and D.‐H.Y.; resources, D.‐H.Y. and C.‐C.L.; writing – original draft preparation, T.‐Y.C.; writing – review and editing, D.‐H.Y. and T.‐A.C.; supervision, C.‐C.L. and C.‐W.K. All authors have read and agreed to the published version of the manuscript.

## Ethics Statement

The study was conducted according to the guidelines of the Declaration of Helsinki and approved by the Institutional Review Board of Tri‐Service General Hospital (approval code: T63888).

## Consent

Informed consent was obtained from all subjects involved in this study.

## Conflicts of Interest

The authors declare no conflicts of interest.

## Data Availability

The data that support the findings of this study are available from the corresponding author, D.‐H.Y., upon reasonable request.
